# Influence of Surface Damage on Weld Quality and Joint Strength of Collision-Welded Aluminium Joints

**DOI:** 10.3390/ma18132944

**Published:** 2025-06-21

**Authors:** Stefan Oliver Kraus, Johannes Bruder, Florian Schuller, Peter Groche

**Affiliations:** Institute for Production Engineering and Forming Machines, TU Darmstadt, D-64287 Darmstadt, Germany; stefan.kraus@ptu.tu-darmstadt.de (S.O.K.); johannes.bruder@ptu.tu-darmstadt.de (J.B.);

**Keywords:** collision welding, impact welding, electromagnetic pulse welding, welding window, metallographic weld zone, multi-material design, lightweight concepts, weight reduction, emission reduction

## Abstract

Collision welding represents a promising solid-state joining technique for combining both similar and dissimilar metals without the thermal degradation of mechanical properties typically associated with fusion-based methods. This makes it particularly attractive for lightweight structural applications. In the context of collision welding, it is typically assumed that ideally smooth and defect-free surface conditions exist prior to welding. However, this does not consistently reflect industrial realities, where surface imperfections such as scratches are often unavoidable. Despite this, the influence of such surface irregularities on weld integrity and quality has not been comprehensively investigated to date. In this study, collision welding is applied to the material combination of AA6110A-T6 and AA6060-T6. Initially, the process window for this material combination is determined by systematically varying the collision velocity and collision angle—the two primary process parameters—using a special model test rig. Subsequently, the effect of surface imperfections in the form of defined scratch geometries on the resulting weld quality is investigated. In addition to evaluating the welding ratio and tensile shear strength, weld quality is assessed through scanning electron microscopy (SEM) of the bonding interface and high-speed imaging of jet formation during the collision process.

## 1. Introduction

Mobility is a fundamental aspect of modern society, enabling access to education, healthcare, and the labour market. It includes private and public transport, logistics, and infrastructure [1]. In 2023, the transport sector accounted for 21% of global CO_2_ emissions, 38% of which came from passenger cars [2,3,4]. In addition to greenhouse gases, non-exhaust particulate emissions–especially from tyre wear and brake dust–are gaining attention as they are strongly influenced by vehicle weight [5,6,7,8,9,10,11,12,13]. Reducing vehicle mass, therefore, helps to reduce both CO_2_ emissions and particulate pollution such as microplastics [14,15,16]. This underlines the environmental importance of lightweight design [17].

These lightweighting goals can be achieved by using high-strength aluminium alloys, which are commonly used in automotive engineering [18]. However, joining these alloys using conventional fusion-based welding techniques presents significant challenges. These include the formation of a heat-affected zone (HAZ) leading to strength degradation, porosity caused by stable oxide layers, distortion due to the high thermal conductivity of aluminium, and hot cracking associated with alloying elements [19,20,21,22]. To exploit the lightweight potential of high-strength aluminium alloys, a joining method is required that mitigates these challenges, most of which are directly attributable to the thermal input inherent in conventional welding processes.

In this area, the solid state welding or collision welding process group opens up new production potential. The joining mechanism is based on the application of sufficiently high pressure to join similar and dissimilar material combinations without active heat input. Therefore, the formation of intermetallic phases in the joint zone is avoided [23,24,25]. Joining feasibility is defined by collision velocity and angle, with the process window categorised into solid-phase, liquid-phase, and hybrid joining regions [26]. Electromagnetic pulse welding (EMPW) facilitates collision welding in industrial settings by accelerating a joining partner via a strong electromagnetic field, creating a high-speed impact weld [25,27,28,29].

Due to transient effects, both the collision velocity and the collision angle change dynamically during the EMPW collision process, directly influencing the properties of the resulting joint [30,31]. Currently, EMPW process design is carried out exclusively in an iterative manner, which involves considerable time and cost expenditure and leads to uncertainties regarding both joint performance and process stability [32]. The existing iterative process design is based on idealised “optimal” conditions with cleaned and undamaged material surfaces. However, such conditions do not always exist in industrial environments.

Fine scratches on component surfaces can result from upstream manufacturing processes such as rolling or extrusion. In addition, handling, transport, and storage of semi-finished products are particularly relevant as potential sources of surface damage in the form of deeper scratches [33]. The correlation between the sensitivity of the collision welding process to environmental influences-demonstrated in previous studies, for example, through varying surface roughness [34]. The occurrence of scratches in the production process suggests that such scratches, under otherwise identical process parameters, may lead to fluctuating joint strengths or even to a complete failure of the weld. Such a condition is undesirable from a quality assurance perspective in industrial manufacturing processes and is unacceptable due to the potentially high degree of process uncertainty.

There are two basic approaches to solving this problem, both of which require an assessment of the component surfaces.

1. Use of exclusively undamaged/scratch-free components:

This approach requires considerable effort in terms of handling and storage of the semi-finished products within the production process. In addition, it leads to high rejection rates, as even components with minor damage from previous process steps must be sorted out.

2. Identification and evaluation of critical scratches:

In this approach, only components with scratches deemed critical are discarded, or alternatively, the process parameters—such as collision velocity—are adjusted so that a sufficiently strong joint is achieved even in the presence of non-critical scratches.

The second approach significantly reduces the rejection of components and semi-finished products and allows for less complex concepts for component handling and storage, as only the exceeding of critical scratch characteristics needs to be prevented. This contributes significantly to cost reduction and resource savings, as the need for semi-finished products, packaging protection materials, and complex control measures is reduced.

In the context of this study, a model test rig for collision welding is used to investigate the influence of scratches on joint quality systematically. In this process, scratch depth, number, and orientation relative to the welding direction are varied. The aim is to identify critical thresholds that will define the minimum surface quality requirements that must not be undershot by manufacturing, handling, and storage processes. This enables the targeted selection of suitable process routes in an industrial setting with minimal effort. The experimental setup, as well as the tests and analyses conducted, are detailed in the following section.

## 2. Materials and Methods

The experimental procedure is divided into three process steps. First, the specimens are prepared. Subsequently, they are subjected to collision welding under defined collision angles and collision velocities using the model test rig. After the collision welding tests, the specimens are analysed using various measurement techniques to evaluate the joint formation and the resulting weld quality.

The specimens to be welded (collision area: length × width: 12.5 mm × 12 mm) are taken from AA6060-T6 extruded flat material (flyer, thickness: 2.0 mm, tensile strength R_m_: 215 N/mm^2^) and AA6110A-T6 extruded flat material (target, thickness: 2.0 mm, tensile strength R_m_: 290 N/mm^2^) by shear cutting. The materials’ chemical composition corresponds to DIN EN 573-3 [35]. The proportions of the alloying elements are listed in Table 1. For each material, three tensile tests are performed on a Zwick Roell 100 combined tensile (Ulm, Germany) and compression testing machine to determine tensile strength in accordance with DIN 50125 [36]. The samples are produced so that the extrusion direction of the material is parallel to the welding direction.

**Figure 1:**
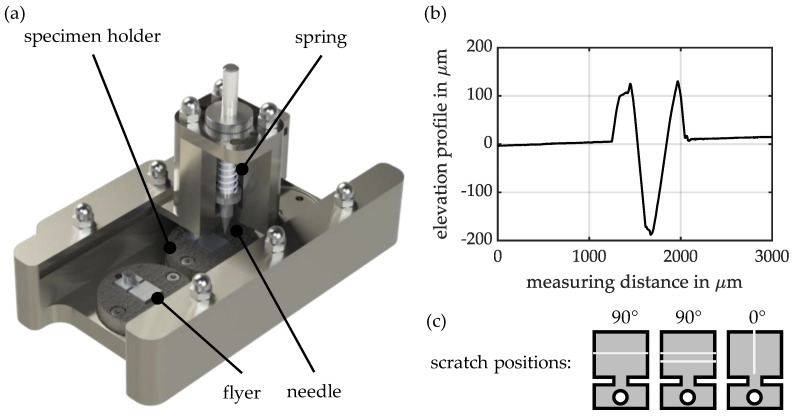
(**a**) The experimental configuration of the scratching device for perpendicular scratches. (**b**) Height profile of a scratch on a prepared specimen (scratch depth 3). (**c**) Representation of the scratch designs to be investigated.

The sample preparation is carried out depending on the specific experimental configuration. Smooth, undamaged samples are cleaned with acetone before the test and visually inspected for surface defects. If the influence of scratches is to be specifically investigated, these are manually introduced into the samples. The introduction of the defined scratches is carried out using the device shown in Figure 1a. In this case, a spring-loaded, hardened needle with a tip angle of 30° is guided over the sample surface. The device allows for precise adjustment of the needle protrusion and scratch depth.

Through flexible positioning of the samples in special fixtures in the device, it is possible to rotate the samples so that scratches can be applied parallel, perpendicular, or at an angle (e.g., 45° rotation) to the welding direction. Additionally, the samples can be offset laterally to allow multiple parallel scratches to be introduced on the sample surface at a distance of 4 mm from each other. In this study, the influence of a scratch parallel to the welding direction and one or two scratches perpendicular to the welding direction is investigated, as shown in Figure 1c. The scratches are introduced into the surface of the AA6060-T6 (flyer) in the used aluminium-aluminium material combination, as this alloy, due to its lower strength, experiences scratches more quickly in industrial production environments compared to the harder AA6110A-T6. Due to its lower strength, AA6060-T6 is used as the flyer in the experiments, as this reduces the stress on the flyer rotor when the predetermined breaking point is torn (see [37]). Furthermore, the selection of the lower-strength material as the flyer allows for better transferability to future scratch investigations with multi-material combinations (e.g., aluminium-steel, and the EMPW process). On the one hand, when using a combination of aluminium and steel in the EMPW process, aluminium is always used as the flyer material. This is due to its much higher electrical conductivity compared to steel [38]. On the other hand, aluminium will always show a higher sensitivity to scratches.

The determination of the scratch depth is carried out using the tactile roughness measurement device Hommel Etamic-T 8000 (Villingen-Schwenningen, Germany). The height profile of the prepared samples is recorded with two equidistant measurement lines perpendicular to the direction of the scratch. The scratch depth is then extracted from the height profile and averaged over the two measurements. Figure 1b shows an example of the height profile of a sample prepared by scratching to the greatest examined depth at a 90° orientation. The scratch of the specific sample has an indented depth of 190 µm. The needle scratches the sample surface, displacing material and forming elevations on both sides of the scratch. In the sample shown, these elevations are 125 µm high. The ratio of the height of the side elevations to the actual scratch depth remains almost constant across different scratch depths. In the following sections, scratch depth is defined as the depth of the scratch in relation to the zero line.

Table 2 summarises the scratch parameters investigated in this study and their corresponding designations. For each parameter combination, three specimens are welded and examined. The combinations are differentiated based on the number of scratches per specimen (scr), scratch orientation (0°: parallel to the welding direction; 90°: perpendicular to the welding direction), and scratch depth (d). The scratch depth is divided into three ranges: d1 (15–30 µm), d2 (50–80 µm), and d3 (170–200 µm). The division into ranges is made because, due to the fluctuating material thickness (±0.05 mm) and the necessary clearance of the scratch device, there is always some variation in the introduced scratch depth. The comparatively large scratch depths are selected to investigate potential surface damage that can occur during industrial processes, such as vibration feeders or dropping materials into transport boxes without protection. The wide range of depths is used to determine the maximum depth up to which welding with good strength values is possible.

**Figure 2:**
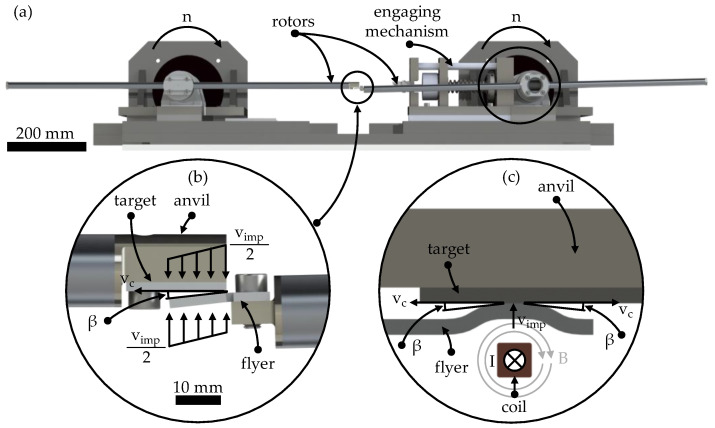
(**a**) The model test rig consists of two synchronised rotating rotors and an engaging mechanism. (**b**) The specimens are mounted at the end of each rotor. (**c**) Process setup EMPW according to [39].

The collision welding process is carried out on the institute’s model test rig for collision welding, which is shown in Figure 2a. Two specimens—the so-called flyer and the so-called target—are accelerated to a defined collision velocity v_imp_ using rotating drives and brought into collision at a predetermined collision angle β. The collision angle is set by bending the flyer, while the collision velocity is adjusted through the motor speeds. A detailed description of the test rig can be found in source [37].

The detailed view (Figure 2b) shows the two specimens immediately before impact. Additionally, the characteristic process parameters—collision velocity v_imp_, collision point velocity v_c_, and collision angle β—are highlighted. The collision point velocity can be calculated using Equation (1), where the collision velocity v_imp_ is the velocity of the specimen perpendicular to the flyer [26].

(1) $$\tan\beta =\frac{v_{imp}}{v_{c}}$$

During the collision, a rolling motion of the flyer occurs with the collision point velocity v_c_ on the rear-supported target, which leads to joint formation between the two joining partners through the formation of a jet. This process forms the basis of joint formation in all collision welding techniques, including the previously mentioned EMPW. In contrast to EMPW with transient collision conditions (where collision velocity and angle vary over the course of the collision, see Figure 2c, the main feature of the test rig is the ability to precisely adjust and maintain constant process parameters over time, as well as the good observability of the process.

By systematically varying the collision angle and velocity, a characteristic process window for the material pairing is determined, which provides insight into the parameter space in which a solid-state connection is possible. The collision velocity v_imp_ is varied in six steps: 279 m/s, 297 m/s, 305 m/s, 314 m/s, 331 m/s, and 349 m/s. High-speed optical observation is performed using an HSFC Pro image intensifier camera from PCO (Kelheim, Germany) and a Milvus Macro 100 mm f2.0 macro lens from Zeiss (Oberkochen, Germany). The camera allows exposure times of <20 ns and the acquisition of up to eight images per collision. The brightness of the high-speed images is ensured by a CAVILUX Smart illumination laser from Cavitar (power: 400 W, wavelength: 640 nm, Tampere, Finland).

**Figure 3:**
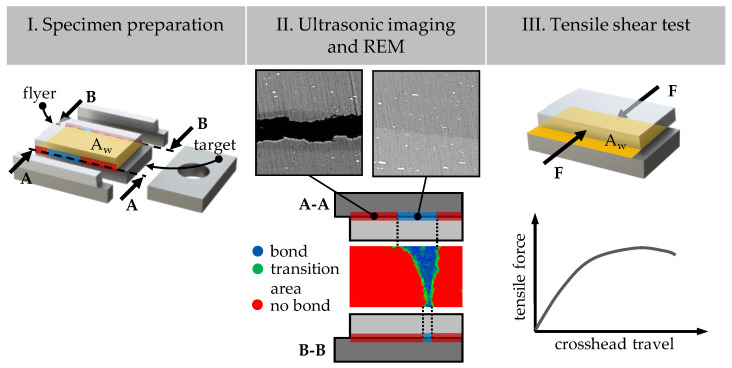
Scheme of the subsequent specimen analysis procedure.

The procedure for the subsequent sample analysis is shown in Figure 3 and is divided into three consecutive steps.

I. Sample Preparation

For the analysis of the welding zone, the edge areas and the mounting surface of the welded samples are first cut off using a wet-cutting machine. The purpose of this is to remove any possible manufacturing influences in the edge areas due to the specimen preparation and to ensure a defined and fully overlapping joining surface between the flyer and the target. The reason for this is that due to process tolerances of the test rig, a precise 100% overlap between the flyer and target cannot be guaranteed. By trimming to a width of 8 mm ± 1 mm and a length of 14 mm ± 1 mm, a standardised/comparable sample geometry is ensured. Subsequently, the lateral separation surfaces of the samples are planed on both sides with SiC paper (grit sizes P320, P400, P600, P800, P1200, P2500, and P4000) and polished with diamond suspension (3 µm and 1 µm) to enable precise analysis of the joining zone.

II. Ultrasonic examinations and SEM

The welding ratio is determined using an ultrasonic measurement system of the type MiniScanner from the manufacturer Amsterdam Technology (Zwinderen, The Netherlands). Its probe captures a measurement area in the form of a long slot, measuring 25.0 mm in length and 11.4 mm in width, with a combined rotational and translational movement. The scanner’s recording provides a graphical representation of the welded (blue) and non-welded (red) areas, as well as the transition zones (green) within the samples. The welding ratio, the ratio of the welded area to the overlapping area A_w_, is analysed using a Matlab script. Therefore, the transition area is also divided into welded and non-welded areas using stored threshold values for the ultrasonic signals. The accuracy and reliability of the ultrasonic imaging results in this study are validated by comparing them with metallographic cross-sectional images. The exact nature of the transition zone is still not fully understood and remains a subject of further investigation. The microscopic imaging of the cross-sectional images of the weld zone is carried out using a Phenom ProX Scanning Electron Microscope (SEM) manufactured by Phenom-World (Waltham, MA, USA), as well as an MIRA3 SEM from TESCAN (Brno, Czech Republic).

III. Tensile Shear Test

To determine the shear tensile strength of the welded specimens, they are clamped into a specially designed test setup and then loaded on a Zwick Roell 100 combined tensile and compression testing machine at a test speed of 0.1 mm/s until separation occurs. A detailed description of the test setup and the operation of the device can be found in source [26]. The shear tensile strength is calculated from the previously measured overlapping bonding area A_w_ and the measured tensile force. This enables a comparable assessment of the mechanical joint quality. The repeatability of the tensile shear tests depends on the overall Zwick Roell 100 combined tensile and compression testing machine with an integrated force sensor.

## 3. Results

### 3.1. Welding Process Window

**Figure 4:**
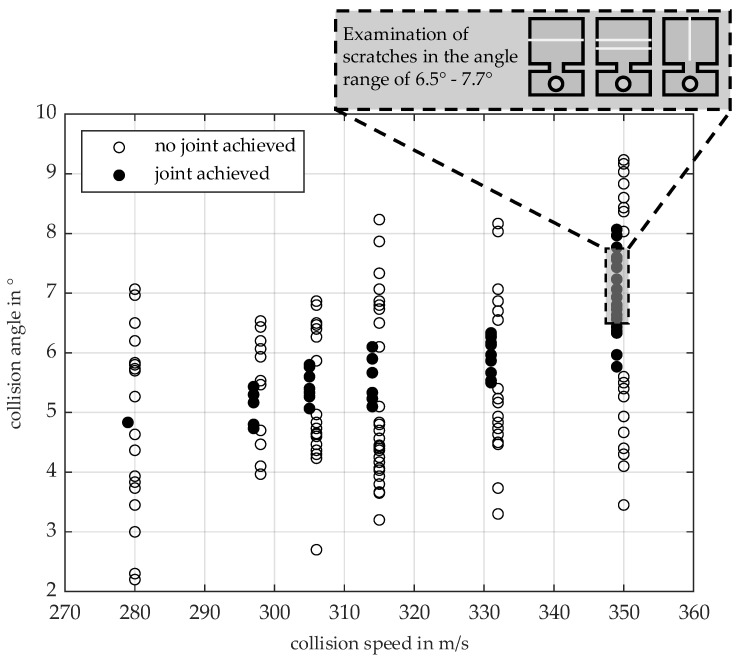
Welding process window of the material combination AA6110-T4 (target, thickness: 2.0 mm) and AA6060-T6 (flyer, thickness: 2.0 mm).

Figure 4 shows the experimentally determined process window for the investigated material combination of AA6060-T6 (thickness 2.0 mm) and AA6110A-T6 (thickness 2.0 mm) in the velocity range up to a maximum of 349 m/s and collision angles between 2.0° and 9.5°. The first joints are observed at a collision velocity of 279 m/s and a collision angle of 4.8°. A collision welding test is classified as a joined test if manual separation of the specimens is not possible after the test (black dots). To improve the clarity of the diagram, the unsuccessful tests are shifted 1 m/s to the right of the successful tests on the collision velocity axis (white dots).

As the collision velocity increases, the angular range within which a connection is formed between the joining partners increases. A key parameter for describing this range is the so-called limit angle, which describes the minimum or maximum collision angle at which a joint is still possible at a given velocity. The observed increase in the range in which joints occur with increasing velocity has been documented several times in the literature, see [40,41]. The behaviour of the lower limit angle varies depending on the material combination, see [37,40,41]. For certain material combinations, such as aluminium-steel, a decrease in the lower limit angle with increasing collision velocity was even observed. On the other hand, the upper limit angle shows an increasing curve for all material combinations investigated so far. In the present investigations, a comparatively strong increase in the lower limit angle with increasing velocity was observed, significantly more pronounced than in previous studies. The angle range of the process window at 297 m/s lies between the lower limit angle of 4.7° and the upper limit angle of 5.5°. At a maximum collision speed of 349 m/s, this results in an angle range of 5.8° to 8.4° for the material combination investigated. The physical reasons for the sharp increase in the lower limit angle with increasing speed are not yet fully understood.

Since the joint quality typically deteriorates as the limit angles are approached, and since the exact setting of the collision angle on the test bench cannot be fully guaranteed due to dynamic effects and tolerance-related deviations (see [37]), an intermediate angle range of 6.5° to 7.7° at 349 m/s was selected for the following investigations of the influence of scratches on joint formation. This choice makes it possible to observe the influence of scratches in an isolated manner under constant and reproducible process conditions.

The area marked in Figure 4 represents the three scratch configurations to be investigated in the described process window area. The complete test plan with all investigated parameter variations is summarised in Table 1.

### 3.2. Welding Ratio

**Figure 5:**
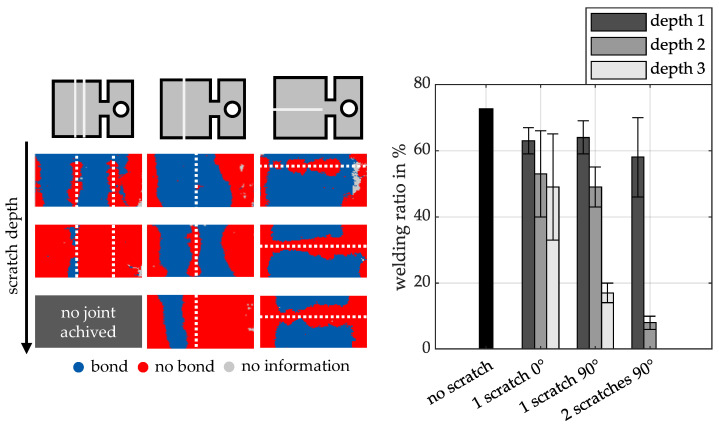
(**left**) Ultrasonic examination of the scratched and welded specimens (welding direction: from right to left). (**right**) Welding ratios of the analysed specimens.

The results of the ultrasonic examinations of the scratched samples are shown in Figure 5. The left side of the figure shows the qualitatively analysed ultrasonic images of exemplary samples of the respective test configurations. The dotted lines indicate the position of the scratches. The scratch depth increases towards the bottom for all scratch variants.

Areas where the two materials are joined are coloured blue. Red areas indicate areas that are not joined. It is clear that the bonded area decreases significantly with increasing scratch depth in all the configurations analysed. This effect is particularly pronounced for scratches with an orientation of 90° to the welding direction. In the configuration with two parallel scratches and maximum scratch depth, no joint could be achieved within the angle and velocity range considered.

The bar graphs on the right show the quantitative percentage welding ratios derived from the ultrasonic measurements. The welding ratio is defined as the ratio of the welded area to the total overlap area A_w_ (see Figure 3). For comparison, the welding ratio of a reference sample without scratches and with identical process parameters is also shown.

The results show that scratches with an orientation of 0° to the welding direction have only a moderate effect on the welding ratio compared to the scratch-free reference sample with increasing depth (reduction of 12% at the lowest depth and 24% at the greatest depth). In contrast, the welding ratio decreases significantly more with a 90° scratch orientation, up to 56% at the greatest scratch depth. At the lowest scratch depth, the welding ratio for the 0° and 90° orientations is in the same range. However, increasing the number of scratches has an even more significant effect. Even at the lowest depth, two parallel scratches of identical depth and orientation result in a reduction in welding ratio of 25% compared to the reference sample. At a medium scratch depth, the welding ratio is only 8%. At the greatest scratch depth, as already mentioned, no joint is achieved.

**Figure 6:**
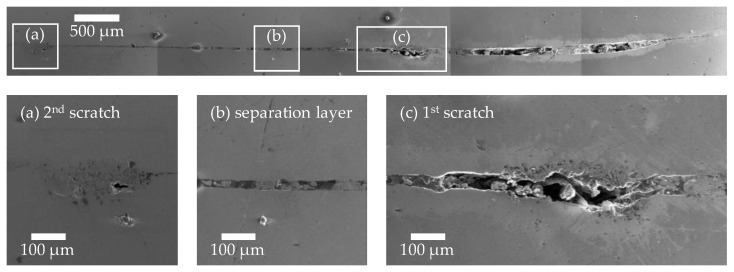
Panoramic view of the weld zone of a specimen with two medium-depth scratches oriented at 90° to the welding direction (on top: target, on the bottom: flyer, welding direction: from right to left, panoramic view stitched together from five separate SEM images). Magnification sections: (**a**) 2nd scratch, (**b**) separation layer with inclusions, (**c**) 1st scratch.

Figure 6 shows a panoramic view of the weld zone of a specimen with two 90° scratches of medium-depth, composed of individual SEM images. The weld direction is analogous to the ultrasonic images shown above, from right to left. The SEM panorama confirms the ultrasonic image. In the area before the first scratch, the whole area is not connected and is characterised by a thick separation layer with inclusions. After the first scratch, the thickness of the separating layer decreases, but the samples are not connected along the length up to the second scratch. In the area of the second scratch, a more porous structure can be observed compared to the base material. The original scratch is completely filled with this microstructure. Small parts or remnants of this porous structure can also be seen in the area of the first scratch. After the second scratch, a connected area can be identified.

### 3.3. Tensile Shear Test

**Figure 7:**
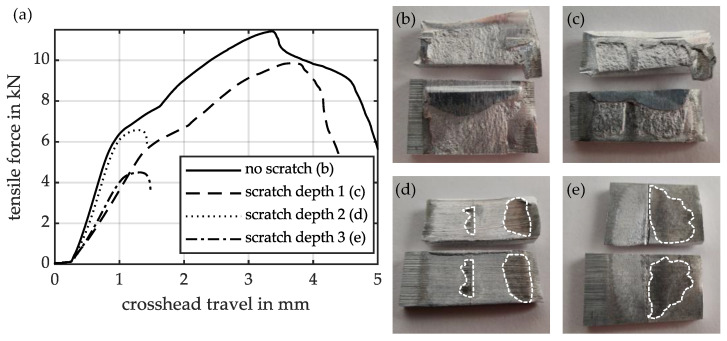
(**a**) Force-travel curves of specimens with and without scratches in the tensile shear test. (**b**–**e**) Images of the separated specimens after the tensile shear test (on top: flyer, on the bottom: target, welding direction: from right to left).

Figure 7a shows examples of force-travel curves obtained when determining the tensile shear strength of a scratch-free specimen and of specimens with a scratch oriented at 90° to the welding direction, at three different scratch depths. The curves show the maximum load and travel distance before the joint separates. The ratios of the force values determined do not correspond exactly to the shear strength values of the specimens presented later, since the cutting of the welded specimens is subject to the tolerances described, and therefore, deviations in the maximum force occur to a certain extent. When calculating the tensile shear strength, the determined force is related to the overlapped area A_w_. The specimen with the lowest scratch depth already shows a slightly lower endured maximum tensile shear force compared to the scratch-free specimen. As the scratch depth increases, the maximum tensile shear force decreases further, and the welded specimens separate after the testing machine has travelled a shorter distance. A significantly steeper drop in tensile shear force is also observed in specimens with medium and maximum scratch depths. Previous studies [37] have identified two distinct failure mechanisms in tensile shear tests on collision-welded specimens. Those with high tensile shear strength values fail due to shear in the lower-strength base material, i.e., the AA6060-T6 of the flyer in the present material combination (no scratch and scratch depth 1). In contrast, specimens with low tensile shear strength values (scratch depths 2 and 3) fail due to sliding in the weld zone. This sliding after reaching the maximum bearable force occurs almost abruptly, so that the switch-off threshold of the testing machine is reached, as the specimens can no longer transfer any force from this moment on.

The right-hand side of the figure shows examples of completely separated specimens after the tensile shear test. The scratch-free reference specimen (Figure 7b) demonstrates shear failure in the base material over almost its entire length. Only at the beginning and end of the joint does sliding occur in the weld zone (light silver, smooth areas). In the specimen with the lowest scratch depth (Figure 7c), shear failure in the base material also occurs over the largest area. However, sliding in the weld zone is already recognisable in a small partial area near the scratch. At medium scratch depth (Figure 7d), the area in which sliding occurs in the weld zone is predominant. Dark discolouration (white outlined areas) in the area of the scratch indicates that the jet remains there for a longer period of time, or even becomes trapped. At the greatest scratch depth (Figure 7e), dark discolouration (white outlined areas) is observed across almost the entire surface of the weld zone in front of the scratch. Only in the area after the scratch are light, silver-coloured, smooth areas visible, indicating failure due to sliding in the weld zone. Comparing this with the previously shown ultrasonic evaluations reveals that the dark discolourations are present in areas where no joint is formed. The ultrasonic images of the three samples shown in Figure 7c–e, which have a 90° oriented scratch in three scratch depths, are shown in the centre column of Figure 5. Comparing the welded and non-welded areas of the separated specimens with the ultrasonic images reveals a high degree of correspondence.

**Figure 8:**
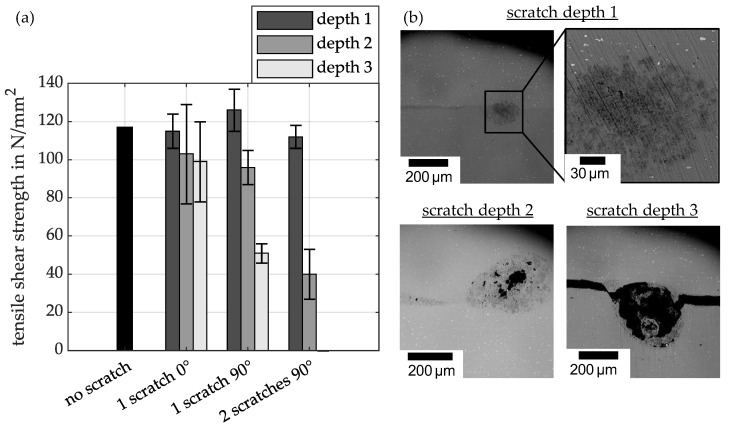
(**a**) Tensile shear strength of the analysed specimens. (**b**) SEM images of the scratch zones at the three different scratch depths.

Figure 8a shows the tensile shear strength of the specimens that were previously analysed using ultrasonic imaging. As can be seen, scratches with a shallow depth (15–30 µm) have no significant influence on the strength of the joint. This applies both to a scratch oriented at 0° to the welding direction and to one or two scratches oriented at 90°. In accordance with the results of the quantitative welding ratios (see Figure 5), the tensile shear strength decreases from the average scratch depth (50–80 µm) in all configurations investigated. The influence of scratch orientation is clearly recognisable in these results. While a moderate decrease in strength can be observed with scratches oriented at 0°, a 90° orientation (i.e., perpendicular to the welding direction) leads to a significantly greater decrease. The lowest strengths occur in samples with two scratches at a 90° angle and a medium scratch depth. With two scratches at the greatest depth, bonding is not achieved.

SEM images of the scratch zones are taken prior to tensile shear testing. Figure 8b shows an example of the structures observed by SEM for a 90° scratch at three different depths. At low scratch depths, only a locally altered microstructure is visible in the immediate vicinity of the scratch. In the welding direction, both before and after the scratch, the joint is fully welded. At a medium scratch depth, the porous structure, characterised by cavities in the area of the scratch, indicates vortex formation. This suggests turbulence in the escaping jet and entrained material, as well as complex material rearrangements. With a diameter of over 300 µm, the swirled area is significantly greater than the original scratch depth. This means that material melts directly around the scratch during the collision. This structure is also observed in the area directly in front of and behind the scratch, but to a lesser extent. The SEM image of the deepest scratch shows residues of possible vortex formation during the collision. However, compared to the medium scratch depth, there is almost no material in the scratch, and the localised melting around the scratch is smaller in relation to the original scratch depth. Additionally, the materials in the areas before and after the scratch are not connected. The areas on the left and right edges of the scratch in the image suggest that the samples may have been temporarily connected before being separated by a crack. Regarding the SEM images and observations, it should be noted that they are exemplary, as they only permit localised observation within the weld zone. It is not possible to observe the entire length of the scratch.

### 3.4. High-Speed Images

**Figure 9:**
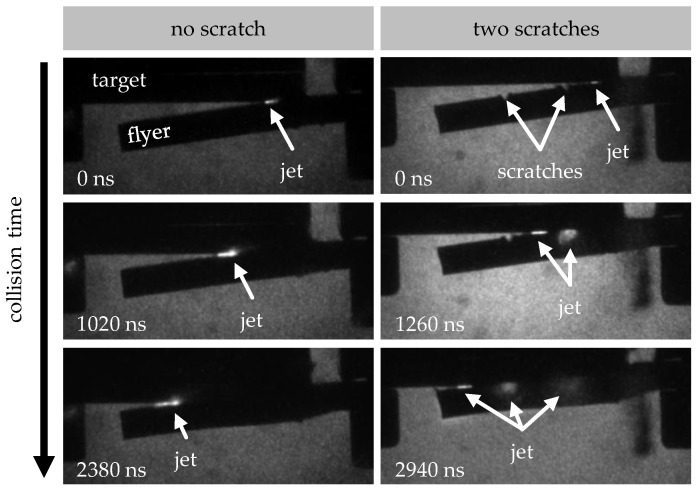
High-speed images taken during the collision of a specimen at the model test rig (welding direction: from right to left, v_imp_ = 349 m/s). (**left**) Specimen with no scratches. (**right**) Specimen with two scratches of scratch depth 2 oriented at 90°.

Figure 9 shows an example of the high-speed images taken during the collision. The images compare a scratch-free reference specimen with a specimen prepared with two medium-depth scratches oriented at 90° to the welding direction. The resulting jet is clearly visible at the beginning of the collision in both sequences of images. The scratches in the prepared specimen are clearly visible in the first image. As the collision progresses, the untreated specimen exhibits the characteristic formation of the jet. This spreads continuously throughout the collision process as the collision gap closes, leaving the collision gap along the collision direction at the end. In contrast, lateral jet clouds form in the specimen with the two scratches. These form in the area of the scratches, or are deflected there from the jet’s original direction of travel. Although a jet is also present in the collision direction, its character and intensity are significantly reduced compared to the reference specimen. The middle image sequence, in particular, shows that the jet is more intense and focused in the non-scratched specimen.

An additional specimen is produced to investigate this phenomenon in more detail. In this specimen, the bore of the target for mounting on the rotor is offset by 1.0 mm laterally in order to deliberately create a lateral offset between the colliding specimens during the collision. This specimen is welded using the same process parameters as before (v_imp_ = 349 m/s, β = 7.2°). A dark, localised deposit similar to the discolourations shown in Figure 7 can be seen in the area of the scratch. This confirms the assumption, based on the high-speed images, that the jet emerges from the side of the sample in the area of the scratches.

## 4. Discussion

The results presented demonstrate how different scratch configurations influence the quality of joint formation during collision welding of two aluminium specimens made from AA6060-T6 and AA6110A-T6 alloys. The effects of scratches of different depths and orientations relative to the welding direction on the welding result are investigated. These scratches lead to varying welding ratios and interlayers, directly affecting the resulting tensile shear strength.

The main reason for the observed differences in strength values is the direct correlation between the welding ratio and the calculated shear strength. As the latter is related to the complete overlap area A_w_ (see Figure 3), reducing the welded proportion (with constant strength per surface element) will result in a drop in the calculated tensile shear strength. This relationship is clearly illustrated in Figure 5 and Figure 8. It can be seen that shallow scratches with a depth of 15–30 µm only slightly reduce the strength values compared to scratch-free reference samples. The lowest strengths are observed for deep scratches at an angle of 90°.

Previous studies have shown that a jet consisting of removed oxide layers, impurities, and ionised material, together with high surface pressure, is essential for forming a high-quality joint. The jet both cleans and activates the surfaces. [34] Based on the results obtained in this study, it can be assumed that scratches above a certain depth negatively affect the formation and propagation of the jet, thereby reducing the welding ratio. This assumption is supported by the high-speed images in Figure 9, which show disturbed jet formation, and the cross-sections in Figure 6 and Figure 8. Two characteristic scenarios can be distinguished.

1. Jet inclusion in the scratch: As the collision progresses, the jet hits the scratch and becomes part of it. Depending on the size of the scratch, it is either completely or partially filled. The resulting heat, which has also been observed in collision welding tests at low collision angles [37], can cause the material to melt locally. Combined with the removed material, this creates a pronounced intermediate layer. Similar findings were reported in EMPW welding processes involving ground surfaces [42]. In particular, structures running perpendicular to the welding direction lead to increased interlayer formation, which can be attributed to jet inclusions.
2. Jet interruption due to scratch geometry: In some cases, the jet is deflected or interrupted so strongly by the scratches (the indentation and the elevations at the edges of the scratches, see scratch profile in Figure 1) that there is insufficient energy left for surface cleaning and activation. SEM images in Figure 8 show vortex structures indicating such jet disturbance. Notably, this prevents joint formation in large areas before the scratch, whereas a joint is formed after the scratch. This suggests strong turbulence in the closing collision gap, hindering the jet and connection formation in the area in front of the scratch. The turbulent atmosphere escapes from both sides of the collision gap via the scratch, creating atmospheric conditions again after the scratch that enable joint formation.

Compared to the 90° scratches described above, those in a 0° orientation have less influence on jet and joint formation. With these scratches, the sample is fully joined in the areas outside the scratch area, regardless of the depth of the scratch. However, in the scratch area, there is almost no joint formation for the three scratch depths. This may be due to a lack of material being pressed into the 0° oriented scratch during the collision. The local plastic deformation and the local material flow are not sufficient to form a joint. On the other hand, for a 90° oriented scratch, the possibility of material being pressed into the scratch is greater due to the rolling motion of the flyer on the target surface during the collision. Nevertheless, some joints do occur in certain areas at low scratch depths, due to sufficient plastic deformation and local material flow within the scratch area. This clearly demonstrates the sensitivity of the process to external influences. At higher scratch depths, there are no joined areas within the scratch path. From a certain depth onwards (compare ultrasonic examination in Figure 5), it is no longer possible to smooth out the scratches through the collision process. This results in the complete elimination of joint formation in these areas.

## 5. Conclusions

This study determines and analyses the process window of the material combination AA6060-T6 (extruded flat material, thickness: 2.0 mm) and AA6110A-T6 (extruded flat material, thickness: 2.0 mm) using a model test rig for collision welding. Based on this, the influence of different types of scratches on the joint quality of collision-welded samples is analysed systematically. The scratches vary in terms of depth, orientation, and number. The results clearly demonstrate that increasing both the scratch depth and number leads to a significant reduction in the tensile shear strength that the joint can endure. Scratches oriented perpendicular to the collision direction have a particularly critical effect as they lead to turbulence in the jet from a critical scratch depth of 50–80 µm, which is accompanied by a significantly greater reduction in tensile shear strength. This is mainly due to a reduction in the welding ratio. The influence of partially formed intermediate layers on tensile shear strength could not be quantified in this study, so this is an important area for future research.

In addition to further investigating the varying increases in limit angles with different material combinations within their respective welding process windows, future research should analyse the influence of scratches on the resulting intermediate layers more in-depth. As part of an extended parameter study, the influence of other scratch configurations should also be considered to develop a comprehensive catalogue of influencing parameters for industrial applications. This will enable critical threshold values to be identified with regard to scratch depth and number for specific applications. This will enable targeted countermeasures to be derived in the area of material handling, as well as improved control of surface-related quality fluctuations. These measures can be adapted as required, ultimately reducing effort and costs in the industrial production process.

## Figures and Tables

**Table 1:** The chemical composition of the aluminium alloys in accordance with DIN EN 573-3.

Alloy	Alloying Elements								
	Si	Fe	Cu	Mn	Mg	Cr	Zn	Ti	Others
AA6060-T6	0.30–0.60	0.10–0.30	0.10	0.10	0.35–0.60	0.05	0.15	0.10	0.15
AA6110A-T6	0.70–1.10	0.50	0.30–0.80	0.30–0.90	0.70–1.10	0.05–0.25	0.20	0.20	0.15

**Table 2:** Summarised scratch parameters.

Description	Number of Scratches (scr)	Orientation	Depth (d) in µm
1scr-0°-d1	1	0°	15–30
1scr-0°-d2	1	0°	50–80
1scr-0°-d3	1	0°	170–200
1scr-90°-d1	1	90°	15–30
1scr-90°-d2	1	90°	50–80
1scr-90°-d3	1	90°	170–200
2scr-90°-d1	2	90°	15–30
2scr-90°-d2	2	90°	50–80
2scr-90°-d3	2	90°	170–200

## Data Availability

The original data presented in the study are openly available in [43].
